# Supplementary material for: Quantifying and Mitigating the Effect of Preferential Sampling on Phylodynamic Inference
Source: PLoS Comput Biol. 2016 Mar 3;12(3):e1004789. doi: 10.1371/journal.pcbi.1004789 (PMC4777449; doi:10.1371/journal.pcbi.1004789)
Supplement: S1 Appendix — (PDF) [file pcbi.1004789.s001.pdf]

Appendix

Hyperproportional simulations

To explore preferential sampling relationships with greater clustering than direct proportionality, or *hyperproportional* preferential sampling, we also perform a simulation study with $\beta_1 = 2, 3$. Fig. A-1 shows the pointwise statistics, while Table A-1 lists the time interval statistics. The results are largely consistent with $\beta_1 = 1$, but with slightly more bias under the BNPR.

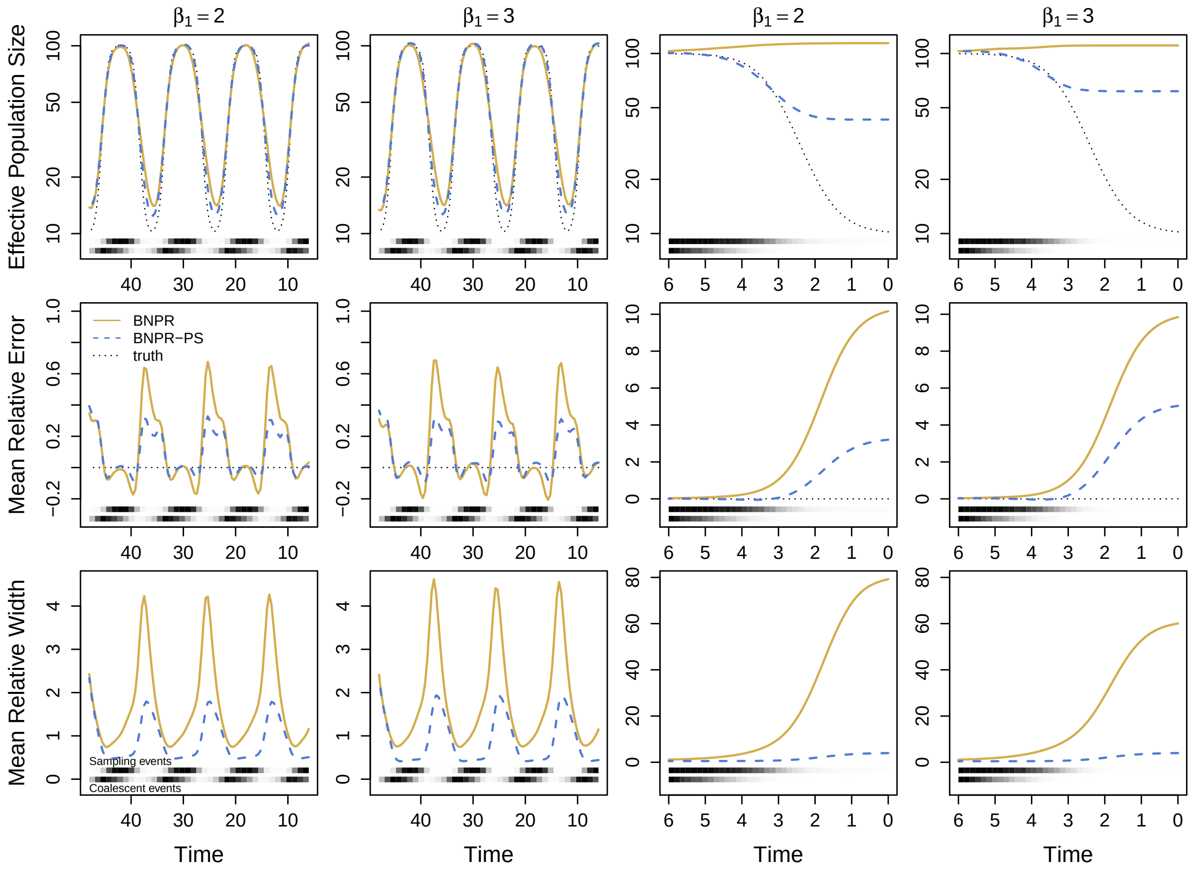

Fig. A-1: Comparison of pointwise statistics with hyperproportional preferential sampling; dotted lines represent the truth, where applicable. Solid yellow lines represent the conditional method BNPR (ignoring preferential sampling), while dashed blue lines represent the sampling-aware method BNPR-PS (accounting for preferential sampling). The first row shows true and estimated effective population sizes, the second shows mean relative error, while the third shows mean relative width of the 95% Bayesian credible interval. The left two columns show the interval $(6, 48)$ where both models perform at their best. The right two columns show $(0, 6)$, where BNPR-PS performs significantly better. At the bottom of each plot, the distribution of sampling events (above) and coalescent events (below) are shown. Time is in months.

	$\beta_1 = 2.0$ $(6, 48)$		$\beta_1 = 3.0$ $(6, 48)$		$\beta_1 = 2.0$ $(0, 6)$		$\beta_1 = 3.0$ $(0, 6)$	
	BNPR	BNPR-PS	BNPR	BNPR-PS	BNPR	BNPR-PS	BNPR	BNPR-PS
MRD	0.240	0.146	0.242	0.147	3.784	0.237	3.839	0.311
MRW	1.865	0.979	1.919	0.938	51.507	1.413	57.701	1.444
ME	0.989	0.987	0.990	0.978	0.852	0.988	0.891	0.972

Table A-1: Averaged time interval summary statistics of the hyperproportional simulations over the interval $(6, 48)$ where both methods perform well, and the most recent interval $(0, 6)$ where BNPR-PS performs considerably better.

Negative control simulations

In the negative control simulations section above, we seek to differentiate between misspecification error due to preferential sampling and error due to few observations (long periods without coalescent events). In Fig. A-2 we simulated sampling times from random piecewise constant sampling intensities independent from effective population size. We generated the sampling intensity trajectories by selecting change points such that the trajectory has a similar number of low-to-high and high-to-low transitions to the seasonal trajectory used in our simulation study, as well as similar amounts of time with high and low intensity. In Fig. A-3 we simulated sampling times from a Gaussian process sampling intensity, also independent from effective population size. We generated the sampling intensity trajectories by selecting Gaussian process realizations that have similar ranges of values and number of transitions to the seasonal trajectory used in our simulation study. Under BNPR, we see relative errors and relative widths less severe than in the case of preferential sampling as we see in Fig. 2, 3, and A-1. However, the performance of BNPR-PS suffers due to the wildly changing ratios of sampling intensity and effective population size.

Parametric simulations

For completeness, we also explored model misspecification in a correctly-specified parametric context. We use an exponential effective population size trajectory $N_e(t) = \exp(a + bt)$, seeking to simulate a growth scenario and a decline scenario. Working backwards in time, perpetual exponential decline is impossible and results in potentially unbounded times to most recent common ancestor (TMRCA). In order to maintain the correctness of our model, we need a values sufficiently small as to result in reasonable TMRCA in our 100,000 genealogies. We also choose b values so that across the sampling window $t \in [0, 10]$ the effective population size will change by a realistic one order of magnitude. Finally, we choose a values that make ranges of the effective population size trajectories in the sampling interval comparable under both growth and decline scenarios. We select $(a, b) = (-\log(10), 0.2)$ and $(-\log(100), -0.2)$. For both uniform and proportional sampling schedules, we simulated 100,000 collections of sampling times between $t = 0$ and $t = 10$, expecting 500 sampling times each collection. We then simulated genealogies for each sampling time collection from the coalescent as in our

**Growth**	$a = -\log(10) = -2.30$		$b = 0.2$	
	Bias	(±2SE)	Bias	(±2SE)
Unif	-0.00216	(-0.00273, -0.00158)	-0.00003	(-0.00012, 0.00008)
Pref	0.00128	(0.00069, 0.00187)	0.00066	(0.00054, 0.00078)

**Decline**	$a = -\log(100) = -4.61$		$b = -0.2$	
	Bias	(±2SE)	Bias	(±2SE)
Unif	-0.00165	(-0.00222, -0.00108)	0.00003	(-0.00006, 0.00013)
Pref	0.00189	(0.00088, 0.00291)	0.00042	(0.00028, 0.00055)

Table A-2: Estimates and confidence intervals for the bias of estimating the parameters of a correctly specified exponential growth/decline model with preferential sampling.

other simulation studies. We applied an exponential growth/decline parametric maximum likelihood method and summarized the results in Table A-2. In both uniform and preferential sampling we see small, but comparable biases in estimates of parameter a. However, estimates of the exponential growth rate parameter b have no detectable bias under uniform sampling, but have small but significant bias under preferential sampling. This verifies that ignoring preferential sampling causes systematic bias, perhaps of small magnitude, in maximum likelihood phylodynamic estimation even under a simple low-dimensional parametric model.

Regional influenza

We examine three of the remaining regions more closely in Fig. A-4 and A-5 and the final three regions in Fig. A-6 and A-7. We see much more pronounced seasonality in the estimated effective population size trajectories produced by BNPR-PS, as well as noticeable improvements in the relative widths of the Bayesian credible intervals. We see three regions with unusual results. Whereas most of the regions show some seasonality under BNPR which becomes more visible under BNPR-PS, India and Southeast Asia both have little seasonality under both methods. Furthermore, South America has little seasonality under BNPR but some seasonality appears under BNPR-PS. We suspect that these results may be due to inclusion of countries with different flu seasonal patterns into this region.

As a final experiment, we explored the variability in our results when we allow for uncertainty in our genealogical inference. Figure A-8 shows the results of running BNPR and BNPR-PS on eight randomly chosen trees from our BEAST run. We see that the inferred effective population size trajectories are quite similar, suggesting the potential for some robustness to uncertainty associated with genealogical inference.

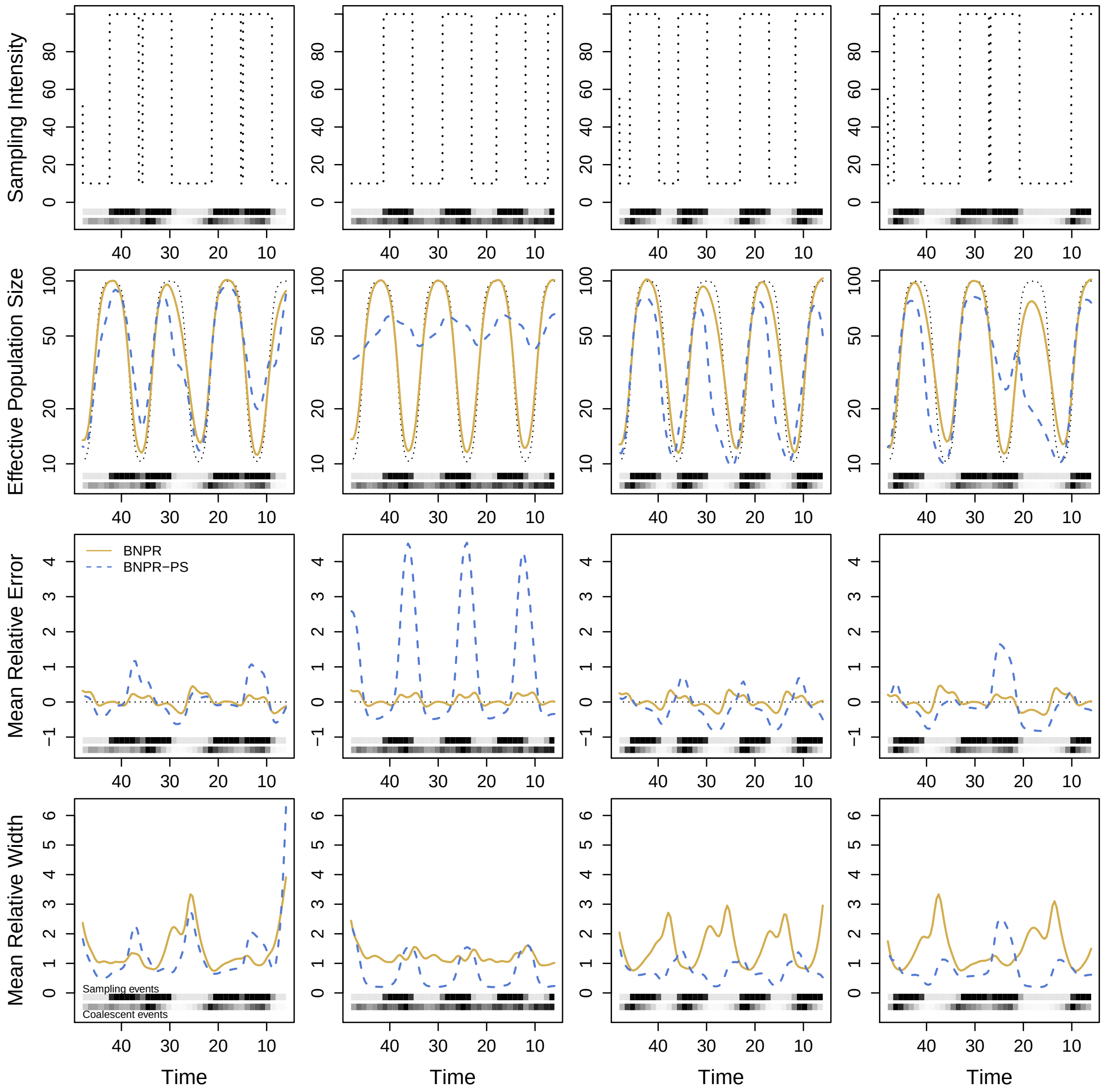

Fig. A-2: Comparison of pointwise statistics for a randomly generated piecewise constant sampling intensity trajectory independent of effective population size. Dotted lines represent the sampling intensity trajectory and true effective population size trajectory. Solid yellow lines represent the conditional method BNPR, while the dashed blue lines represent the sampling-aware model BNPR-PS. The first row shows the sampling intensity, the second shows true and estimated effective population sizes, the third shows mean relative error, while the fourth shows mean relative width of the 95% Bayesian credible interval. The columns represent four realizations of the random sampling intensity trajectory. At the bottom of each plot, the distribution of sampling events (above) and coalescent events (below) are shown.

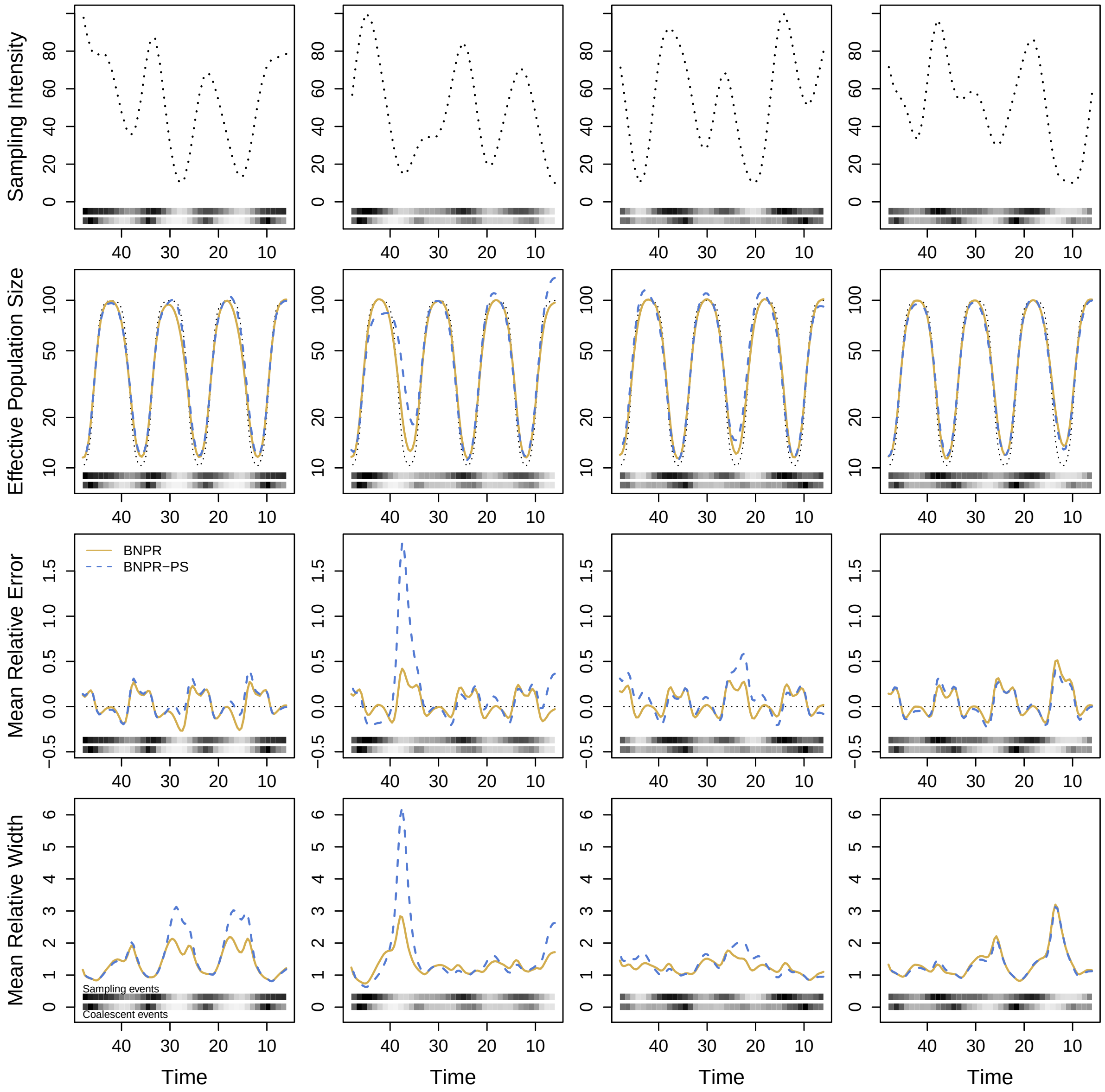

Fig. A-3: Comparison of pointwise statistics for a randomly generated Gaussian process sampling intensity independent of effective population size. Visuals as in Fig. A-2.

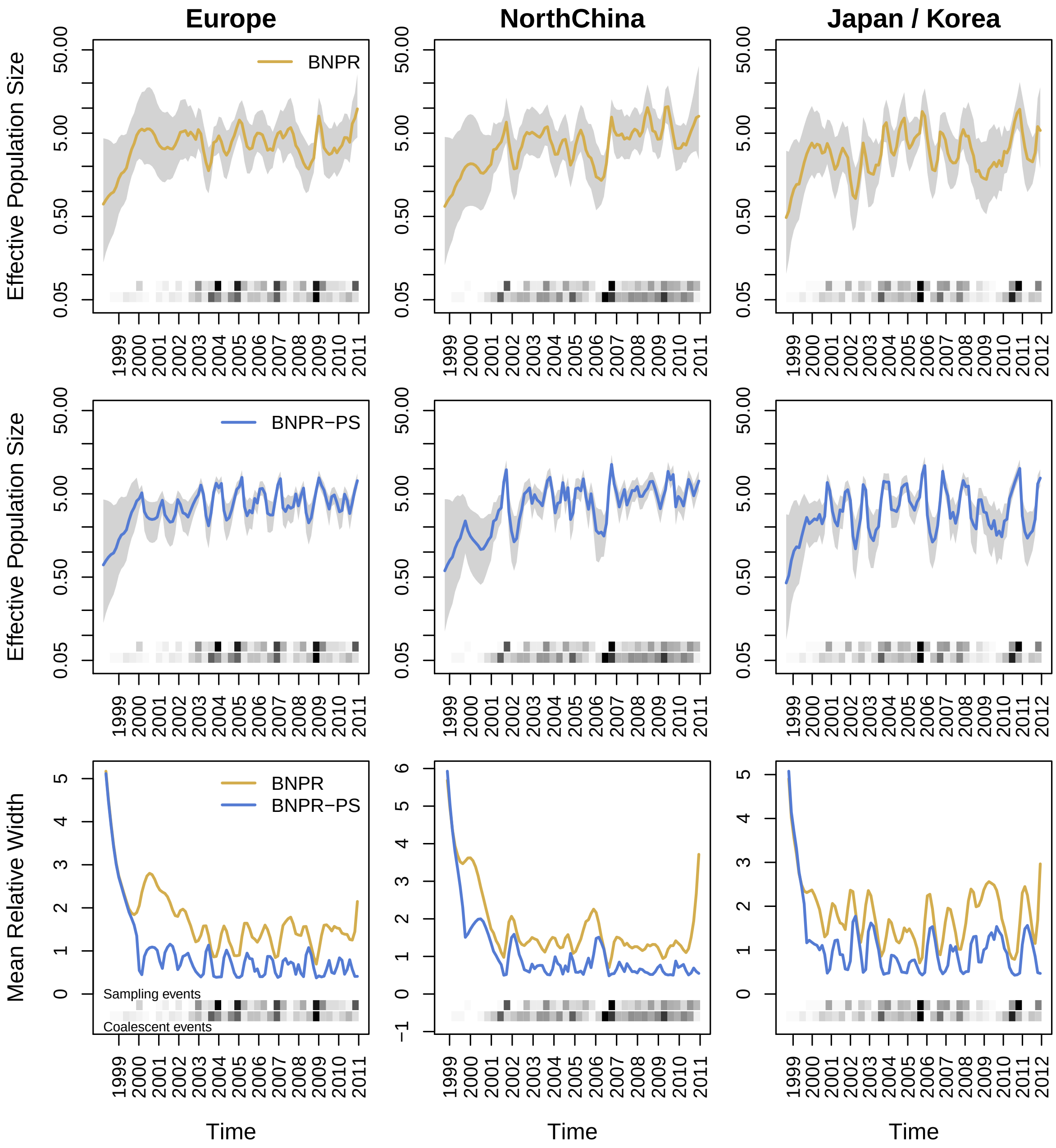

Fig. A-4: BNPR and BNPR-PS models applied to the genealogies inferred from the regional influenza example. We see moderate correlation between effective population size $N^{\gamma}(t)$ and sampling frequencies in the data (Table 2). We see improvements in Bayesian credible interval widths, and BNPR-PS performs as well or better than BNPR everywhere in these examples.

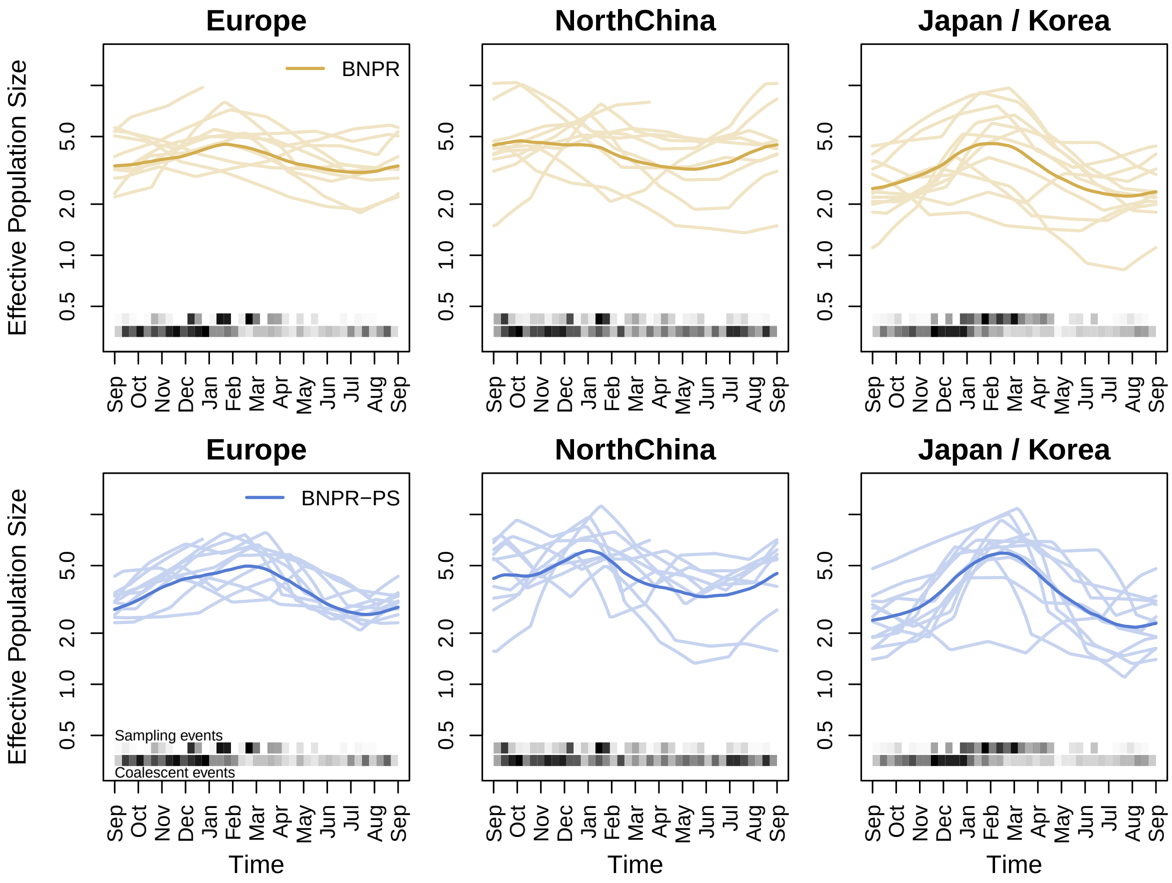

Fig. A-5: BNPR and BNPR-PS models applied to the genealogies inferred from the regional influenza example with years overlaid. We see more pronounced seasonality in the BNPR-PS plots.

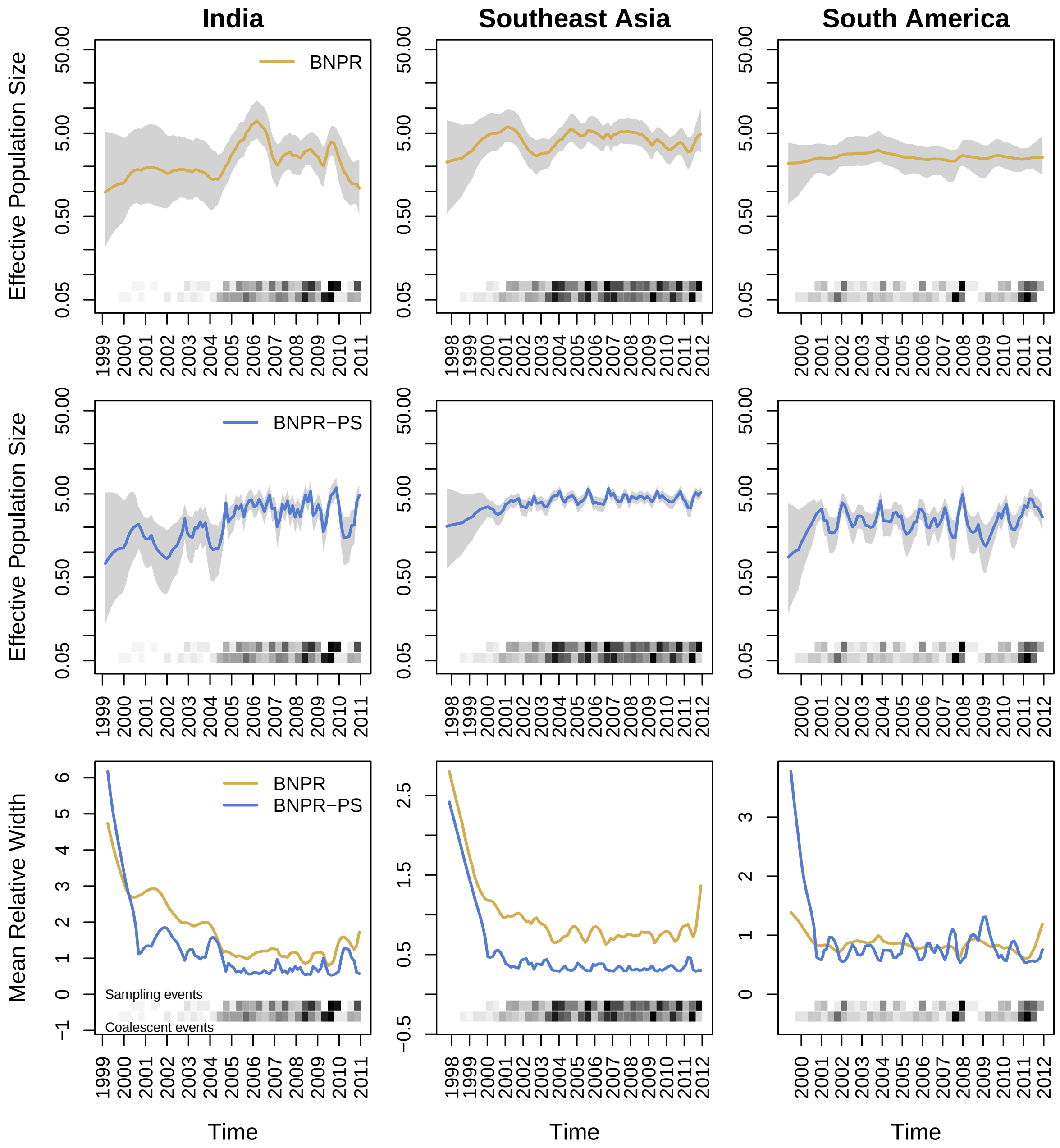

Fig. A-6: BNPR and BNPR-PS models applied to the genealogies inferred from the regional influenza example. Visuals as in Fig. A-4. In South America, we see moderate correlation between effective population size $N^{\gamma}(t)$ and sampling frequencies in the data (Table 2). We see improvements in Bayesian credible interval widths, and BNPR-PS performs as well or better than BNPR everywhere in these examples.

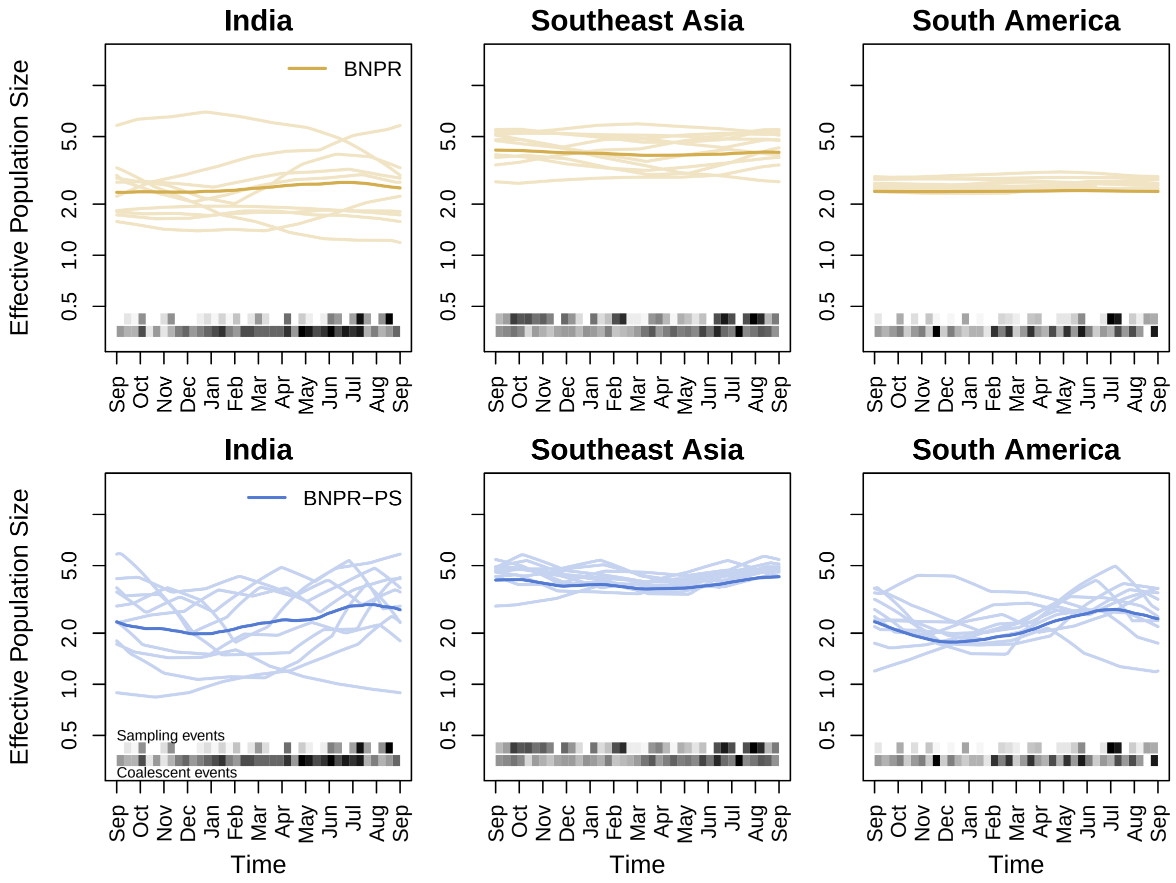

Fig. A-7: BNPR and BNPR-PS models applied to the genealogies inferred from the regional influenza example with years overlaid. We see more pronounced seasonality in the BNPR-PS plots.

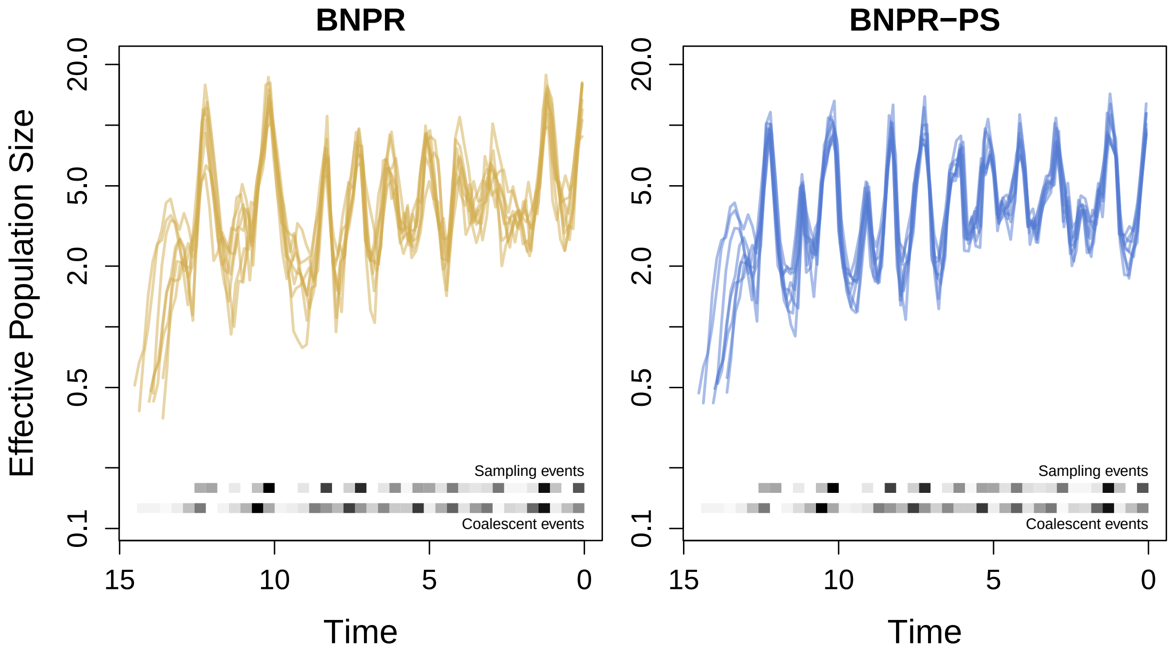

Fig. A-8: BNPR and BNPR-PS models applied to eight randomly selected genealogies from our BEAST inference.
